# A minimally invasive cerclage of the tibia in a modified Goetze technique: operative technique and first clinical results

**DOI:** 10.1007/s00068-021-01857-z

**Published:** 2021-12-24

**Authors:** Stefan Förch, Jan Reuter, Franziska von der Helm, Leonard Lisitano, Christopher Hartwig, Sabrina Sandriesser, Stefan Nuber, Edgar Mayr

**Affiliations:** 1 Department of Trauma, Orthopaedics, Plastic and Hand Surgery, University Hospital Augsburg, Augsburg, Germany; 2 Institute for Biomechanics, Berufsgenossenschaftliche Unfallklinik Murnau, Murnau, Germany; 3 Institute for Biomechanics, Paracelsus Medizinische Privatuniversität Salzburg, Salzburg, Austria

**Keywords:** Minimalle invasive cerclage, Tibial fracture, Operative technique, Clinical results, Complications, Goetze cerclage

## Abstract

**Introduction:**

In spiral fractures of the tibia, the stability of an osteosynthesis may be significantly increased by additive cerclages and, according to biomechanical studies, be brought into a state that allows immediate full weight bearing. As early as 1933, Goetze described a minimally invasive technique for classic steel cerclages. This technique was modified, so that it can be used for modern cable cerclages in a soft part saving way.

**Method:**

After closed reduction, an 8 Fr redon drain is first inserted in a minimally invasive manner, strictly along the bone and placed around the tibia via 1 cm incisions on the anterolateral and dorsomedial tibial edges using a curette and a tissue protection sleeve.

Via this drain, a 1.7 mm cable cerclage can be inserted. The fracture is then anatomically reduced while simultaneously tightening the cerclage. Subsequently, a nail or a minimally invasive plate osteosynthesis is executed using the standard technique.

Using the hospital documentation system, data of patients that were treated with additional cerclages for tibial fractures between 01/01/2014 and 06/30/2020 were subjected to a retrospective analysis for postoperative complications (wound-healing problems, infections and neurovascular injury). Inclusion criteria were: operatively treated tibial fractures, at least one minimally invasive additive cerclage, and age of 18 years or older. Exclusion criteria were: periprosthetic or pathological fractures and the primary need of reconstructive plastic surgery. SPSS was used for statistical analysis.

**Results:**

96 tibial shaft spiral fractures were treated with a total of 113 additive cerclages. The foregoing resulted in 10 (10.4%) postoperative wound infections, 7 of which did not involve the cerclage. One lesion of the profundal peroneal nerve was detected, which largely declined after cerclage removal. In 3 cases, local irritation from the cerclage occurred and required removal of material.

**Conclusion:**

In the described technique, cerclages may be inserted additively at the tibia in a minimally invasive manner and with a few complications, thus significantly increasing the stability of an osteosynthesis. How this ultimately affects fracture healing is the subject of an ongoing study.

## Introduction

Clinical experience and recent research [1] show that elderly patients are unable to follow weight-bearing restrictions after injuries of the lower extremities. In such situations, an osteosynthesis should allow immediate full weight bearing to avoid immobility and resulting complications such as muscle atrophy, pneumonia, or thrombosis. It should also be taken into consideration that 8 weeks of unloading can cause a reduction of bone density up to 1 year thereafter [2]. Under special circumstances (e.g., after suffering multiple injuries), younger patients may also be unable to comply with partial or full weight-bearing restrictions. Prolonged partial weight bearing was recently identified as a risk factor for pseudoarthrosis [3]. It may be assumed that immediate full weight bearing could accelerate the healing process also in monotraumas.

As a result of the foregoing and other considerations, trauma surgeons face the challenge of treating fractures of the lower limb with an osteosynthesis that allows immediate full weight bearing. For proximal femoral fractures, this goal can be achieved routinely using modern operation techniques and implants.

For simple transverse fractures of the tibia treated with nail osteosynthesis, immediate full weight bearing is possible, but not for all spiral fractures, which are more frequently associated with elderly patients. In such cases, mobilization is often restricted to partial weight bearing; thus, modifications of the osteosynthesis allowing immediate full weight bearing are desirable.

A biomechanical study on artificial tibiae recently showed [4] that additional cerclages to a plate osteosynthesis significantly increase the stability: axial stiffness was almost tripled and brought in to the ideal range for fracture healing according to Claes et al. [5]. Shear forces under full weight bearing were significantly reduced and even minor compared to partial weight bearing without cerclage.

**Fig. 1:**
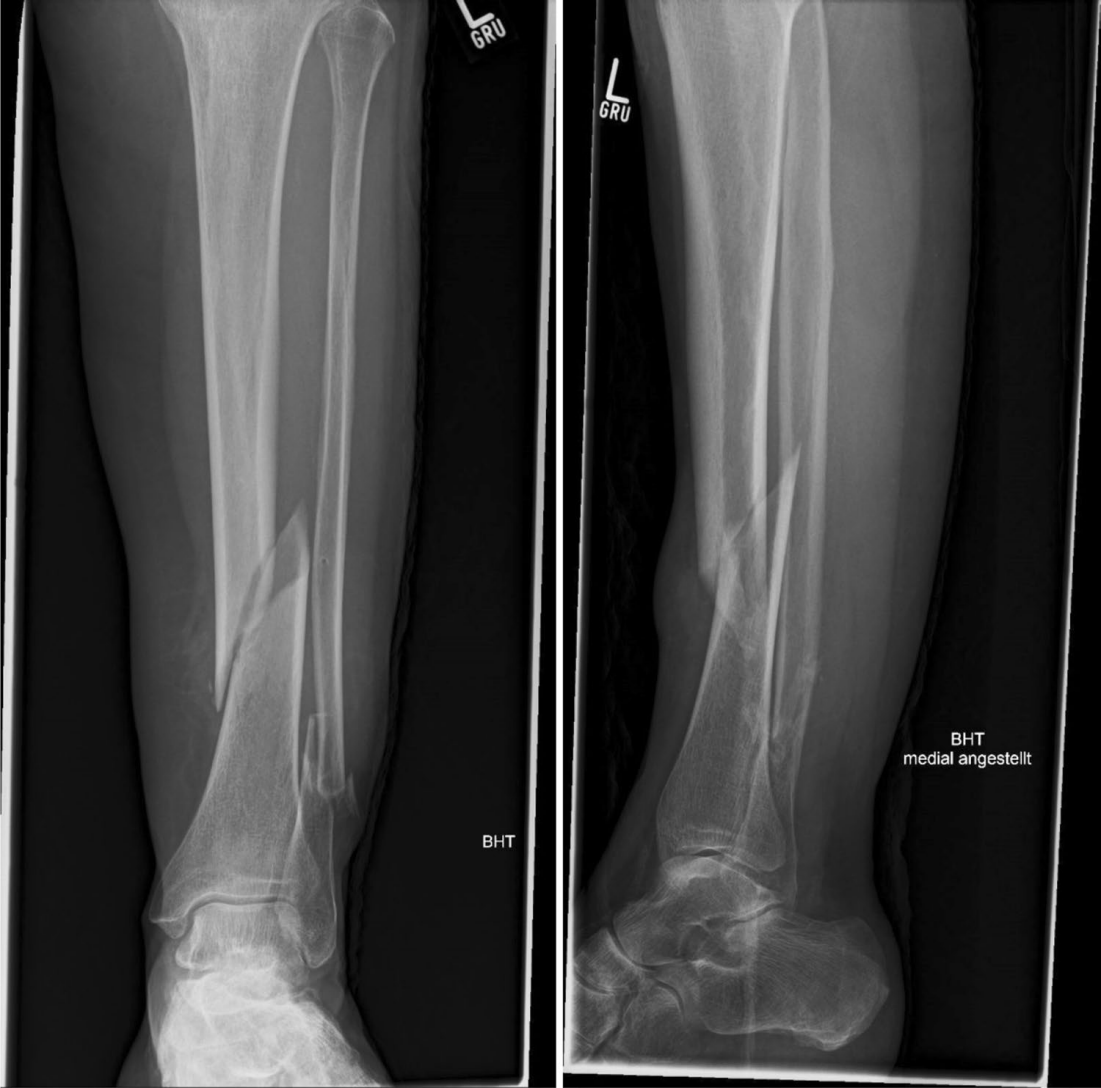
X-ray of a spiral fractue of the lower leg (AO 42A1c/4F2B) in ap and lateral view

**Fig. 2:**
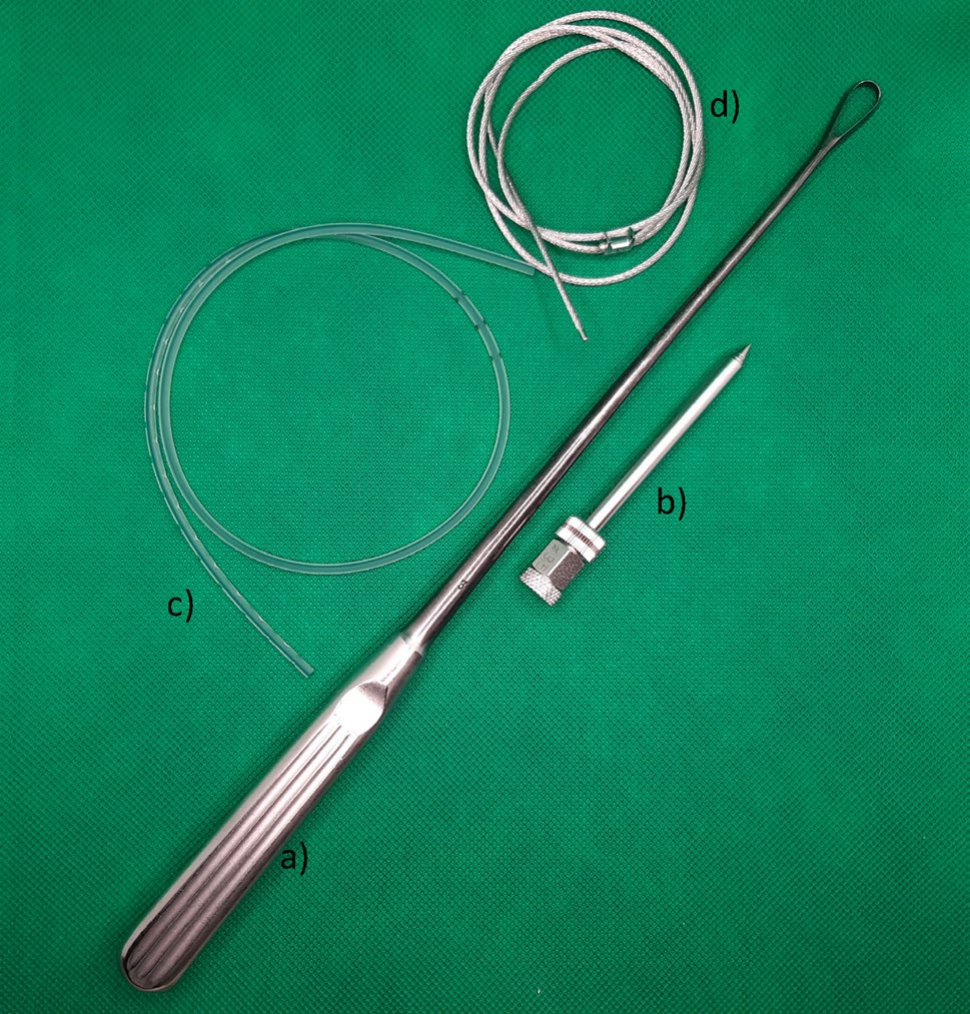
Instruments necessary for the minimally invasive cerclage: **a** curette, **b** tissue protection sleeve, **c** 8 Fr redon drain (REF 21,861 Primed Halberstadt Medizintechnik GmbH, Halberstadt, Germany), and **d** 1.7 mm Cable Cerclage (REF 298.801.01 DePuy Synthes Companies, Oberdorf, Switzerland)

Due to the often critical soft-tissue situation at the lower leg, operations in that region should be minimally invasive. For that reason, a minimally invasive operation technique for the use of cerclages of the tibia was developed. It is based on the technique of Goetze [6] that uses a classical steel cerclage. However, taking into consideration that modern cable cerclages have superior mechanical properties [7], this modified technique is optimized for the use of such modern cable cerclages. This technique is described below and demonstrated in a spiral fracture of the tibia (AO 42A1c, Fig. 1). Figure 2 shows the instruments needed.

## Method

### Surgical technique

**Fig. 3:**
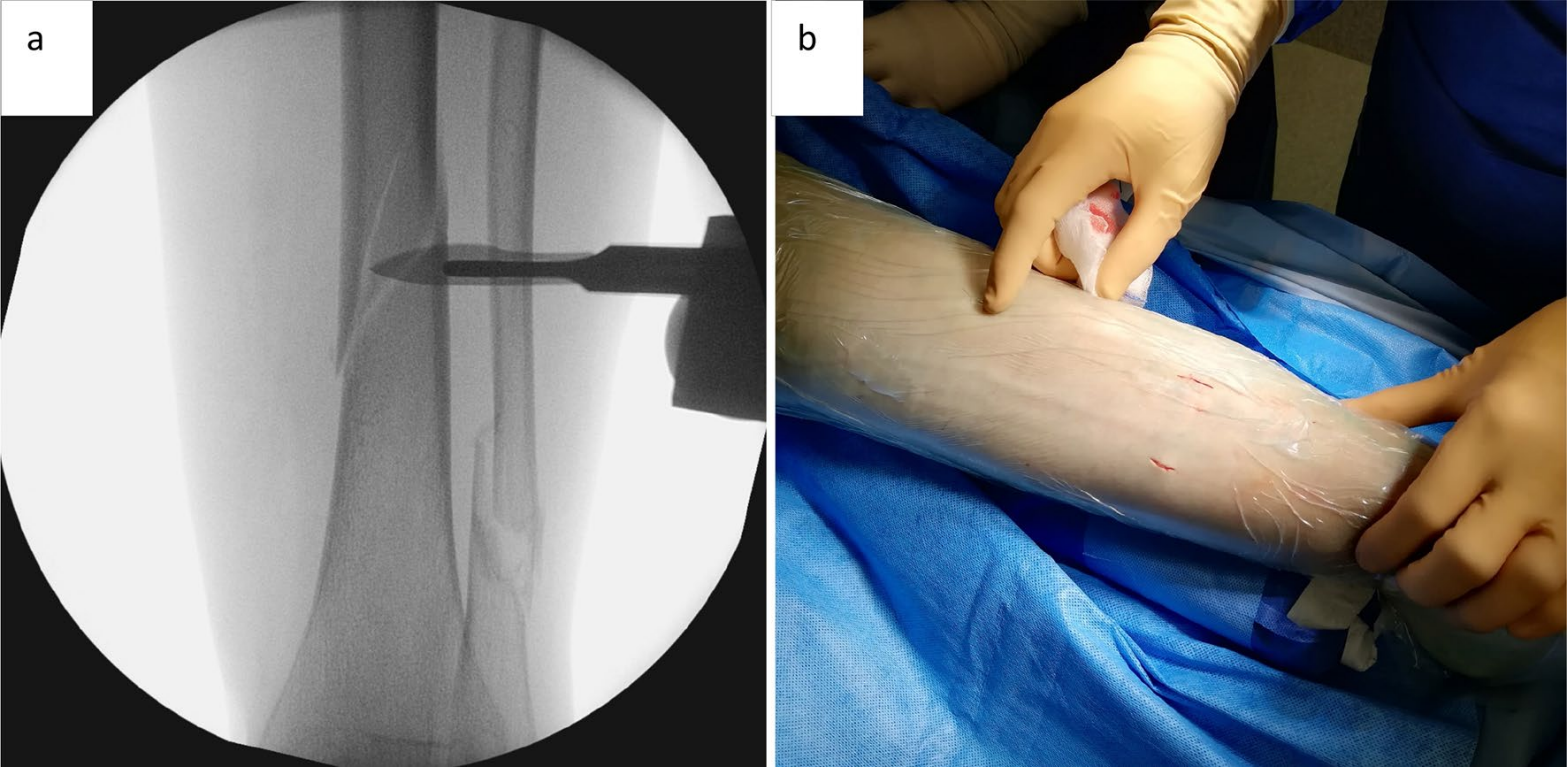
**a** After nearly anatomically reduction of the tibial fracture, the correct position for the cerclage is identified using an image intensifier. **b** Incisions of approximately 1 cm each are set on the anterolateral and dorsomedial edge of the tibia

**Fig. 4:**
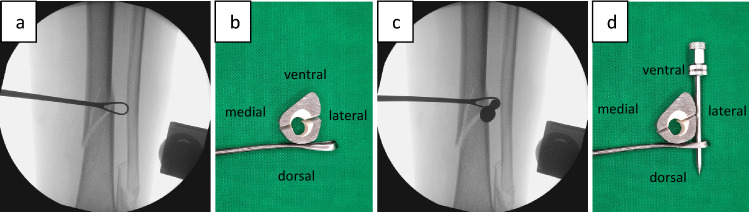
A curette is inserted dorsomedially and pushed to the lateral edge of the tibia under permanent contact to the bone. **a** X-ray in ap view; **b** schematic axial view. The tissue protection sleeve is introduced anterolaterally, slided along the bone, and threated into the loop of the curette. **c** X-ray in ap view; **d** schematic axial view

**Fig. 5:**
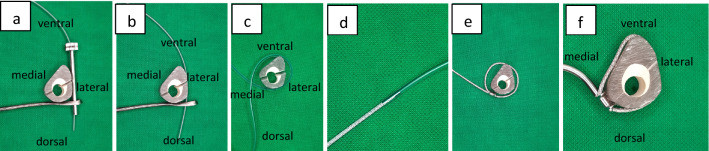
**a** After removal of the trocar an 8 Fr redon drain can be inserted. **b** The tissue protection sleeve can now be removed. The redon drain is threaded into the loop of the curette and can be pulled out through the dorsomedial incision. **c** The drain can be passed along the anteromedial aspect of the tibia using a clamp and is now lying circular around the tibia. **d** The 1.7 mm Cerclage (REF 298.801.01 DePuy Synthes Companies, Oberdorf, Switzerland) fits exactly into the lumen of the 8 Fr drain. **e** The cerclage is inserted via the redon drain and placed circular around the tibia. **f **Schematic axial view of the tibia with inserted and tightened (50NM) cerclage: due to the crimp and the geometry of the tibia, the cerclage has only punctual and no circular contact to the bone

**Fig. 6:**
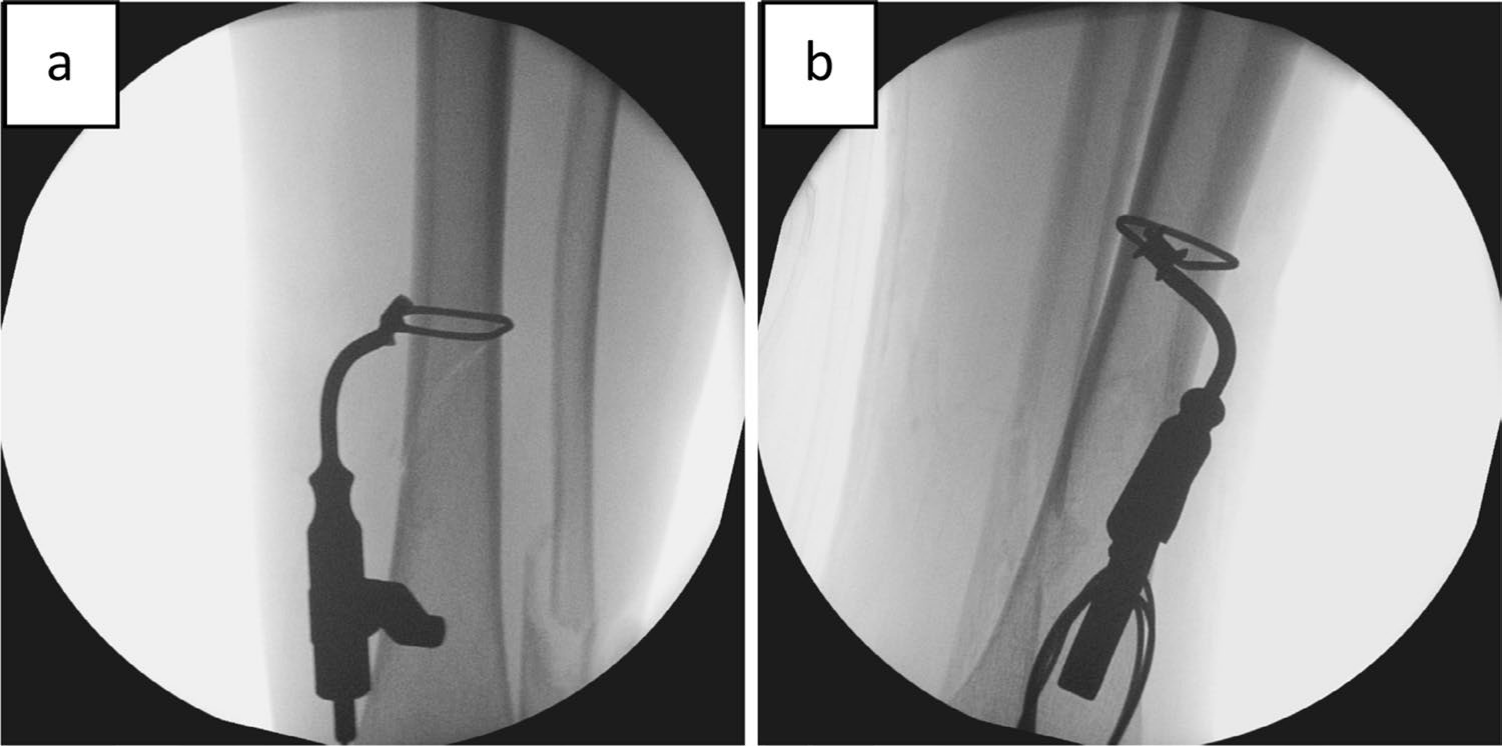
After tightening of the cerclage, the tibial fracture is reduced anatomically (X-ray in ap **a** and lateral **b** view)

In a first step, the fracture should be reduced as anatomically as possible (Fig. 3), an external fixator can be very useful. The correct position for the cerclage is identified via an image intensifier. Two incisions of 1 cm each are placed at the anterolateral and posteromedial aspect of the tibia (Fig. 3). Using scissors, the soft tissue is dissected from the bone, creating a path of 5–7 mm for the cerclage. It is of highest importance, to maintain continuous and close contact to the bone to separate tendons, vessels, and nerves, thereby avoiding encasing them into the cerclage in the following steps. A curette is inserted via the dorsomedial incision and, while maintaining permanent contact to the bone, advanced to the lateral side of the tibia. In the same manner, the tissue protection sleeve of an AO standard fixator is inserted via the anterolateral incision and threaded into the loop of the curette (Fig. 4). This can be done using the image intensifier or under tactile control. After removal of the trocar, an 8 FR redon drain (REF 21,861 Primed Halberstadt Medizintechnik GmbH, Halberstadt, Germany) can be placed via the tissue preserving sleeve. The drain is then threaded into the loop of the curette and may be pulled out through the posteromedial incision. Passing the drain subcutaneously along the anteromedial aspect with a clamp usually does not cause any problems (Fig. 5). After that passage, the drain is now placed around the tibia in a circle. A 1.7 mm Cerclage (REF 298.801.01 DePuy Synthes Companies, Oberdorf, Switzerland) fits exactly into the lumen of the 8 Fr drain and may easily be passed around the tibia. Due to the more robust soft tissue, the lock is normally placed on the dorsomedial edge of the tibia. This position also simplifies the locking of the cerclage. For this procedure, it is sometimes necessary, to extend the dorsomedial incision to 2–3 cm. If a plate osteosynthesis is planned, a position on the medial aspect of the tibia must be avoided. By tightening the cerclage the fracture can be anatomically reduced and primary stability is achieved (Fig. 6). If the fracture is shortened, which is normally the case, a temporary overdistraction using an external fixator can be very helpful. An anatomical reduction enables the bone to bear weight postoperatively and increases the stability of the hole osteosynthesis. After insertion of the cerclage, a nail or plate osteosynthesis (MIPO) may be executed using standard technique.

**Fig. 7:**
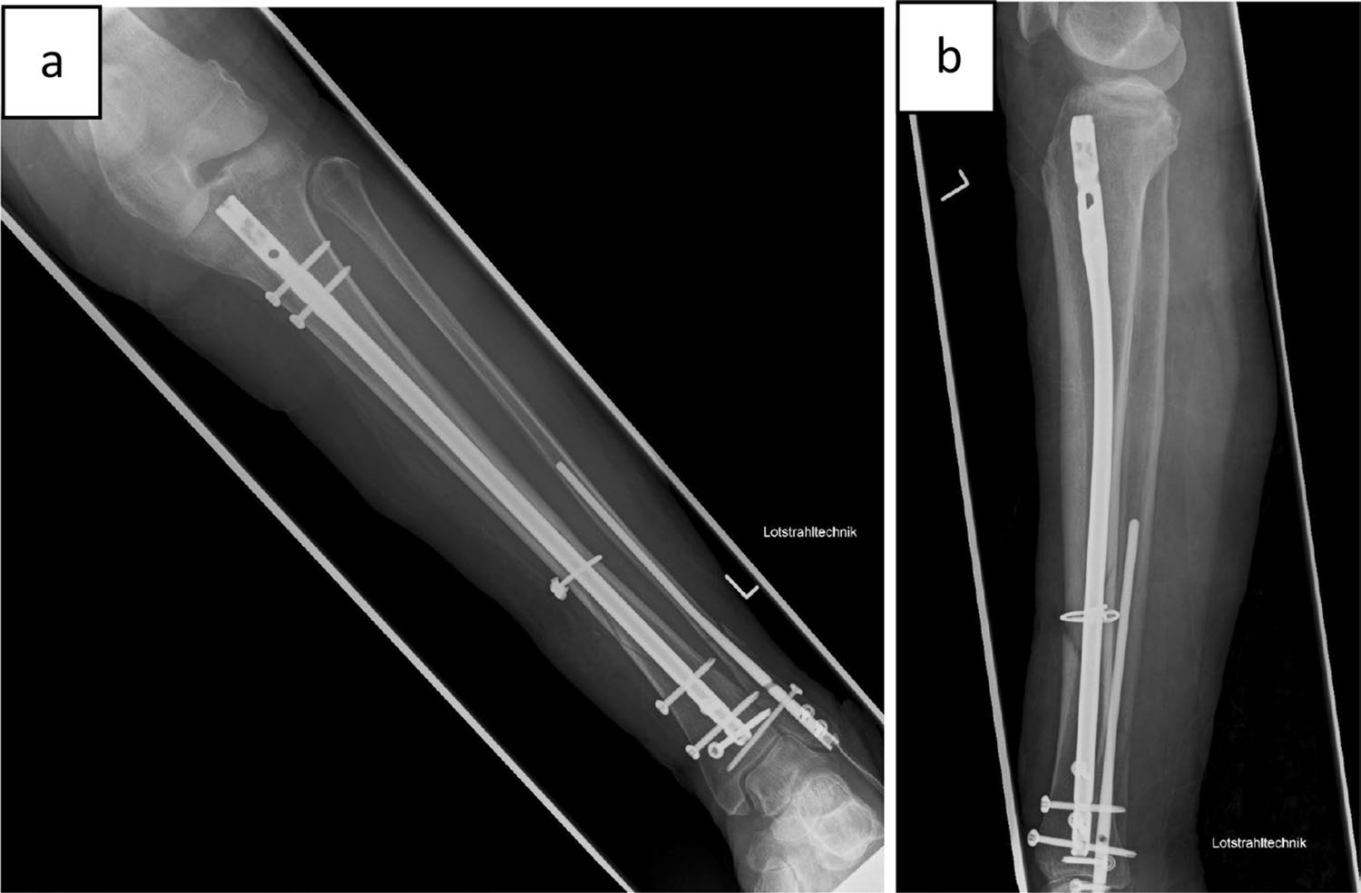
Postoperative X-ray after minimally invasive cerclage and nail osteosynthesis of the tibia (Expertnail, DePuy Synthes Companies, Oberdorf, Switzerland) and fibula (Vitus Fi, Marquardt Medizintechnik GmbH): ap (**a**) and lateral (**b**) view. Mobilization is allowed under full weight bearing

In the given example, a nail osteosynthesis was done for the tibia and the fibula. Full weight bearing was allowed postoperatively (Fig. 7).

### Clinical results

Data from all patients with tibial shaft fractures, which were treated with supplemental cerclages from 01/01/2014 to 06/30/2020, were collected consecutively and analyzed retrospectively using the hospital data system. A randomization took not place. Inclusion criteria were operatively treated tibial fractures, at least one minimally invasive additive cerclage and age of 18 years or older. Exclusion criteria were periprosthetic or pathological fractures and the primary need of reconstructive plastic surgery. Demographic data, type of fracture and operative treatment, postoperative weight-bearing restrictions and complications (such as wound-healing problems, surgical side infections, and neurovascular injuries) were recorded. SPSS was used for statistical analysis.

A positive ethics committee vote (file no. 21–0301) was acquired.

## Results

During the period from 01/01/2014 to 06/30/2020, 96 patients with tibial shaft fractures were treated with 113 supplemental cerclages. Table 1 shows the demographic data; Table 2 the fracture classification according to the AO/OTA classification. 74% (71 patients) received a plate osteosynthesis and 26% (25 patients) received a nail osteosynthesis. An anatomical reduction was achieved in 58 patients (60.4%), a step of half of the thickness of the cortex remained in 26 patients (27.1%), and a step of the full thickness of the cortex remained in 12 patients (12.5%). Additional fibular fractures at the distal third or at the same level as the tibial fracture were treated in 51 cases (53%) with standard osteosynthesis procedures. A plate osteosynthesis was used in 41 cases and a nail osteosynthesis in 10 cases. 51% (49 patients) received partial weight bearing and 49% (47 patients) immediate postoperative full weight bearing.

**Table 1:** Demographic data of the included patients

Number of patients	96
Number of supplemental cerclages	113
age	51,94 years (19–90)
Gender (female vs. male)	42: 54
Side (left vs. right)	37: 59 (39% vs. 61%)
ASA status (I: II: III)	32: 39: 25 (33%: 41%: 26%)

**Table 2:** Fracture classification according to the AO/OTA classification

42A1	52	42A2	9	42A3	1
42B1	0	42B2	9	42B3	4
42C1	0	42C2	8	42C3	7
43A1	1	43A2	1	43A3	0
43B1	1	43B2	1	43B3	0
43C1	2	43C2	0	43C3	0

A postoperative wound infection occurred in 10 patients (10.4%). In 7 of these 10 cases, the infection was located at the medial malleolus and was unrelated to the inserted cerclage. In 3 cases, the infection affected the whole osteosynthesis: there was one superficial impairment of the wound healing over the malleolus medialis and the inserted cerclage, which was treated with debridement and split skin grafting. Implant removal was not necessary. In the other 2 cases, a deep infection required implant removal. Compartment syndromes occurred two times (2%), one directly after the trauma and the other on the second day after treating the tibial fracture with minimally invasive cerclage and plate osteosynthesis on the day of trauma.

Three patients complained of local irritation resulting from the cerclage. After fracture healing and consecutive implant removal, such symptoms disappeared.

In one case, the nervus peroneus profundus was impaired by the cerclage and a hypaesthesia between the first and second toe as well as weakness of the big toe lifter occurred. After early removal of the cerclage, the hypaesthesia and the weakness were reversed.

## Discussion

As early as 1933, Goetze [6] described a technique of a minimally invasive cerclage at the tibia. He used a classic steel cerclage that was inserted using special needles and hooks. A biomechanical comparison proves [7] the superiority of modern cable cerclages. In an artificial bone model, a supplemental cable cerclage significantly increases the stability of the whole osteosynthesis and allows immediate postoperative full weight bearing [4]. The original technique of Goetze was now optimized for modern cable cerclages: in a first step, an 8 Fr redon drain is inserted in a minimally invasive and soft-tissue preserving manner, using a curette and a tissue protection sleeve. Via this drain, the definitive cerclage may be inserted very easily in a second step.

As potential risks of this technique, the additional soft-tissue trauma with consecutive wound-healing problems and infections, the damage of vessels and nerves, and the potential impairment of the periosteal blood supply should be considered.

Using 113 cerclages in 96 patients, a postoperative wound infection occurred in 10 cases, but only 3 (2.7% of the cerclages, 3.1% of the patients) showed a connection to the cerclage. All infections occurred after plate osteosynthesis, resulting in a rate of 14% (10 out of 71). Other studies are reporting infection rates of 9–16% for plate osteosynthesis and 2.9–8% for nail osteosynthesis [8, 9]. In comparison, the infection rate is not increased.

Habernek et al. [10] treated 186 patients with spiral tibial shaft fractures using minimally invasive cerclages and plaster casts. They observed superficial wound infections in 6 patients (3.2%), 2 of them after open fractures. All infections healed after debridement and antibiotic therapy. No increased infection rate could be identified in their cohort.

Tibial shaft fractures generally have a high risk for the occurrence of a compartment syndrome [11–13], especially after high energy traumas. In these cases, a fasciotomy and closed reduction and external fixation are necessary. The observed rate of compartment syndrome (2%) was not increased compared to the rate described in the literature (approximately 11%). The reason for this low rate could be explained by the spiral fracture type suitable for the cerclage, which is normally caused by low energy trauma. The appearance of a secondary compartment syndrome after primary definitive treatment underlines the importance of a careful primary soft-tissue management including the use of primary external fixation and secondary definitive treatment.

Using the minimally invasive technique, the insertion of the cerclage can be executed with very little soft-tissue trauma. Therefore, the risk of additional soft-tissue damage and resulting infections is low and not increased when compared, for instance, with an isolated plate osteosynthesis.

Damage of a nerve (n. peroneus profundus) occurred in one case (0.9% of the cerclages, 1% of the patients). After removal of the cerclage, the defect was to a large extent reversible.

Habernek et al. [10] observed an irritation of n. peroneus in 5 cases (2.7%), caused by local pressure of the cast. There was no damage of nerval structures by the cerclages.

After nail osteosynthesis, neurological complications are reported with rates between 0 and 3% [14], and in single studies, even with 19–30% [14, 15]. Also, larger reviews do not include data concerning neurological complications after plate osteosynthesis [9, 16]. For example, the percutaneous use of reduction forceps can cause neurological damage in 11% [17].

Therefore, the risk of neurological damage through the minimally invasive cerclage is not higher than through isolated nail osteosynthesis or the use of percutaneous reduction forceps.

No vascular injuries were observed, neither by Habernek et al. [10] nor within our group of patients. In the context of an osteosynthesis of the tibia, these adverse events seem to be rare; however, there are existing case reports thereon, especially after nail osteosynthesis [18–20]. Anatomical analyses [21, 22] showed that the arteria tibialis anterior is at risk during the placement of distal locking bolts.

Every surgeon should be aware of the potential vascular damage and the pulse status should be checked and documented at the beginning and at the end of the operation. To avoid vascular damages during the insertion of minimally invasive cerclages, the fracture should be first reduced as anatomically as possible to restore the proper anatomical situation. As spiral fractures are normally caused by low energy traumas, an extreme dislocation of the vessels does not seem very likely. In addition, the correct technique of the operation with preparation while maintaining permanent contact with the bone is essential.

Although the impairment of the periosteal blood supply by cerclages is widely discussed, only a few experimental studies have been performed [23]. An investigation using a fracture model does not exist. Using uninjured [24–26] or osteotomized [27] bones, a preserved blood supply after insertion of cerclages was evidenced several times. A negative impact to the healing of osteotomized bones [27] or the growth of immature bones [28] has not been shown. Furthermore, on a human femur diaphysis, a cerclage covers at maximum 56% of the bony surface, so a circular strangulation is not possible [29]. As the tibial shaft has a more triangular shape compared to the rounder femur, these results should be transferable. The axial view in Fig. 5f shows that the cerclage is not in circular, but only in punctual contact with the tibial bone.

Habernek et al. [30] reported a delayed union in only 4.3% of the patients. All fractures healed by prolonged immobilization. It remains unclear if the delayed union was caused by biological or mechanical reasons. To evaluate the impact of cerclages on the bone healing, further studies will be necessary.

## Conclusion

From a biomechanical point of view [4], a supplemental cerclage at the tibia is suitable to significantly increase the stability of the complete osteosynthesis and may allow immediate postoperative full weight bearing. Using the described technique, a minimally invasive and soft-tissue preserving insertion with low complication rates is possible. The key points of this new technique are the minimally invasive procedure and the anatomical reduction of the bone. This allows the insertion of the cerclage without vascular or nerval affection on one hand, and enables the bone to bear weight on the other hand.

The impact on bone healing of minimally invasive cerclages at the tibia in clinical practice has to be evaluated in further studies.
